# The Theory of the Toxic Origin of Pernicious Anemia

**Published:** 1907-05

**Authors:** 


### THE THEORY OF THE TOXIC ORIGIN OF PERNICIOUS ANEMIA.

---

Austin W. Hollis and Norman E. Ditman describe various experiments that have been carried on in the search for knowledge concerning pernicious anemia. Herter has recently demonstrated the more or less constant presence of certain forms of anaerobic bacteria in the contents of the large intestine. These bacteria can break down proteids into a form which is used by other putrefactive bacteria, among which are the indolforming organisms. Indol occurs quite uniformly in excess in pernicious anemia. Indol produces toxic symptoms in animals whose oxidizing capacity has been decreased. The anaerobic bacteria are limited to the large intestine, where they can be reached by therapeutic measures introduced into the lower end of the gastro-intestinal canal. Irrigation of or local applications in the colon are suggested as a means for their removal. The clinical results of the treatment by colon irrigation in these cases are very happy.—Medical Record, February 2, 1907.

